# An agent-based model of anoikis in the colon crypt displays novel emergent behaviour consistent with biological observations

**DOI:** 10.1098/rsos.160858

**Published:** 2017-04-12

**Authors:** Tim Ingham-Dempster, Dawn C. Walker, Bernard M. Corfe

**Affiliations:** 1 Insigneo Institute for in silico medicine, Pam Liversedge Building, University of Sheffield, Sir Frederick Mappin Building, Mappin Street, Sheffield S1 3JD, UK; 2 Department of Oncology and Metabolism, The Medical School, University of Sheffield, Beech Hill Road, Sheffield S10 2RX, UK; 3 Department of Computer Science, University of Sheffield, 211 Portobello, Sheffield S1 4DP, UK

**Keywords:** agent-based model, colonic crypt, anoikis, homeostasis

## Abstract

Colorectal cancer (CRC) is a major cause of cancer mortality. Colon crypts are multi-cellular flask-shaped invaginations of the colonic epithelium, with stem cells at their base which support the continual turnover of the epithelium with loss of cells by anoikis from the flat mucosa. Mutations in these stem cells can become embedded in the crypts, a process that is strongly implicated in CRC initiation. We describe a computational model which includes novel features, including an accurate representation of the geometry of the crypt mouth. Model simulations yield previously unseen emergent phenomena, such as localization of cell death to a small region of the crypt mouth which corresponds with that observed *in vivo*. A mechanism emerges in the model for regulation of crypt cellularity in response to changes in either cell proliferation rates or membrane adhesion strengths. We show that cell shape assumptions influence this behaviour, with cylinders recapitulating biology better than spheres. Potential applications of the model include determination of roles of mutations in neoplasia and exploring factors for altered crypt morphodynamics.

## 1. Introduction

**Figure 1.**
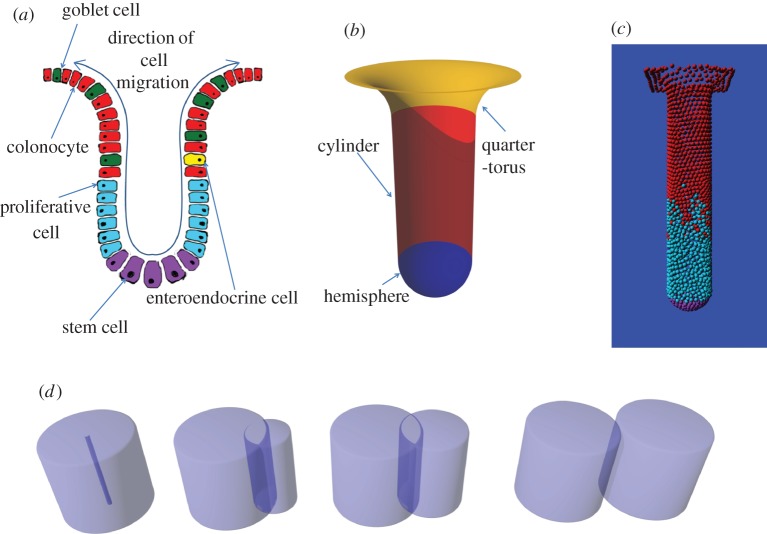
(*a*) Crypts act as cell factories. Stem cells divide at the bottom to provide cells for the proliferating compartment. Cells in the proliferating compartment divide rapidly to provide the various cell types for the constantly refreshing colonic epithelium. (*b*) Visualization of the model crypt membrane. The geometry is modelled as three mathematical shapes, a hemisphere, a cylinder and a quarter torus. (*c*) An image taken from the model during a simulation. (*d*) The process of cell division within the model where a second cell is created, initially with zero radius. As the parent cell shrinks back to its normal volume from the enlarged volume it has at the end of G phase, the new cell expands to the same size and finally detaches. This process takes place through the dividing cell's M phase.

The human large intestine consists of a monolayer epithelium covering a flat mucosal surface punctuated by glands known as the crypts of Lieberkühn (figure 1*a*). These crypts are the functional unit of the colonic epithelium, supporting the rapid turnover of cells in the organ through constant division and differentiation of stem cells located at their base. As such, the perturbation of their turnover and abnormal accumulation of cells is implicated in the formation of colorectal cancer (CRC) [1]. The identification and modelling of underlying processes is therefore crucial to understanding the pathophysiology of disease.

A human colonic crypt contains approximately 2500 cells, or colonocytes [2]. There is a stem cell compartment at the base of the crypt which is estimated to comprise around 10 cells [3]. Cells exiting the stem cell compartment (variously called transit amplifying cells or proliferating cells) account for around 25% of cells in the crypt [4]. Fully differentiated cells include water-absorbing colonocytes which account for roughly 75% of differentiated cells, mucus secreting goblet cells which account for roughly 24% of differentiated cells and hormone secreting enteroendocrine cells which differentiate directly from stem cells rather than from transit amplifying cells and account for less than 1% of differentiated cells [2,5]. There are two main hypotheses concerning the processes governing whether a cell exhibits stem, proliferating, or differentiated behaviour: the *niche* hypothesis suggests that location within the crypt determines cell type, whereas according to the *pedigree* hypothesis cell type is a function of the number of divisions a cell has undergone [6]. Cells migrate from the bottom of the crypt to the top. This movement has been shown to occur even after proliferation stops [7] and to be partially organized by EphB signalling [8,9]. Apoptotic rates are highest at the top of the crypt [10]. A key mechanism of apoptosis within the normal homeostatic epithelium is anoikis [11], whereby loss of contact with the extracellular matrix induces apoptosis. This is known to occur in colonic crypts and its dysregulation is thought to play a role in CRC progression [12] and crypt homeostasis [13].

### 1.1. Computational modelling

A range of computational models have been applied to crypt dynamics, which vary in terms of their basic assumptions, underlying methodologies and degree of spatial resolution. Compartment models consider the composite behaviour of different populations of cells within the crypt. An early compartmental model simulated general tumour growth but was directly applicable to the crypt system [14]. It comprised three compartments, stem cells, proliferating cells and differentiated cells, and implemented rules governing the growth and interactions between these populations in the form of ordinary differential equations (ODEs). Individual cells were not modelled, but rather the total number of cells in each compartment. This model focused on the role of changes to apoptosis and differentiation in tumour formation. It predicted that changes in these processes would lead to increasing plateaus of cellularity before exponential cell growth would occur. The crypt has also been modelled as a continuum [15] using partial differential equations to represent the spatial distribution of the different cell compartments. In this context, cells are still modelled as populations rather than discrete entities. This model was used to investigate the possibility of a neoplastic crypt invading a neighbouring healthy crypt. The model predicted that this type of invasion is a possible mechanism for expansion of the neoplastic tissue.

The first models to consider cells as individual entities were lattice based models—an approach still in use due to its computational efficiency [4]. Here the crypt is modelled as a lattice, or grid, where each lattice point or node can be occupied by a cell. The limitation here is that cells can only move between lattice sites in discrete jumps, meaning intercellular interactions and forces cannot be explicitly considered. The Bravo & Axelrod model [4] focused on calibrating emergent cell turnover rates against known biological parameters and was used to assess various chemotherapeutic protocols from a theoretical viewpoint. Early agent-based models (ABMs) for the first time represented cells as free agents moving on a surface rather than in a fixed grid [16]. These models built on lattice free models of generic epithelial monolayers [17]. Each cell acts as an independent agent with no globally overarching control. This approach is appealing for biological modelling as it captures the nature of biological cells as individual autonomous entities that can respond to signalling cues, and also allows the modeller to capture the important concept of intercellular heterogeneity [18].

Buske *et al.* [19] developed a sophisticated model of the crypt which made a number of predictions about the behaviour of cell populations in the crypt. In particular it showed that cell populations can be regenerated within a crypt even after population extinction. Although this model incorporated the concept of anoikis, the crypt mouth was not included with cells being automatically killed at a certain height from the crypt base.

In most existing models cell death is not explicitly related to a biological mechanism, but there are attempts to capture phenomenologically the known behaviour that cells predominantly die at the top of the crypt. For instance, a deterministic cell death rule is activated as soon as a cell reaches the top of the crypt [16], or cells die stochastically with a probability that increases towards the crypt top [4].

Dunn *et al.* [20] used an agent-based model to examine the role of the basement membrane in crypt formation and also included the concept of anoikis. The model predicted that while anoikis did occur at the crypt mouth, it was also seen throughout the crypt and was most prominent at the crypt base. As such, there was no significant cell migration in the simulated crypt and a rule dictating cell death at the crypt mouth was introduced to give rise to the correct migration behaviour. This could be explained by a putative signalling-related regulatory mechanism at the crypt mouth; however, there is currently no biological evidence for or against such a mechanism.

For a further overview of crypt modelling see Fletcher *et al.* [21].

We sought to develop an ABM in order to explore factors governing cellular homeostasis in the colon crypt, including an explicit representation of the anoikis mechanism. In particular, we hoped to establish whether the anoikis mechanism is sufficient to localize cell death to the crypt mouth and to regulate crypt cellularity without an external control mechanism. Unlike most previous models, we also include a realistic crypt geometry incorporating a section of flat mucosa above the crypt mouth.

## 2. Methods

This model is described using the Overview, Design concepts and Description (ODD) framework [22].

### 2.1. Purpose

The purpose of the model is to investigate the process of anoikis within the colonic crypt. This will be achieved by developing a lattice free model of the crypt similar to previous models [16,23,24]. This model will then be extended to include features which are hypothesized to be important factors in the anoikis process such as the geometry of the crypt top and the attachment force between cells and the basement membrane.

### 2.2. Entities, state variables and scales

Entities in the model represent the cells of the colorectal crypt. Agent state variables are listed in table 1 and global state variables are listed in table 2.

**Table 1.** Per-cell variables, including usage and source where relevant.

variable	use	source
position	cell position	emergent property, initially random
radius	cell radius	calculated from crypt radius and data from the literature [10]
current cycle time	How long has the cell been in the current cycle stage?	emergent property
cycle stage	the current stage of the cell in the model cell cycle	emergent property
growth stage time	length of growth cycle stage for this cell	drawn from a normal distribution with mean of 30 h and s.d. of 2.62 h [10]

**Table 2.** Simulation parameters, including usage and source where relevant.

property	use	source	default value
timestep	number of seconds per timestep	value determined to achieve stable simulation	30 s
G0-phase time	length of time a stem cell spends in quiescence before cell cycle re-entry	value determined from average cycle time (30 h) and number of stem cells biologically expected to be in cycle simultaneously (1 in 10), both taken from the literature [10]	270 h
M-phase time	length of time it takes a cell to divide	calculated from mean cycle time and proportion of cycling cells in M-phase in published literature from biological study [25]	20 min
crypt radius	radius of the vertical portion of the crypt	arbitrary property that all other sizes are calculated relative to	500^a^
crypt height	the total height of the crypt	calculated from cell radius and data in the literature [10]	5918^a^
anoikis threshold	minimum separation required to trigger anoikis	arbitrary value (sensitivity analysis showed no effect on simulation results, see electronic supplementary material)	100^a^
differentiation boundary	position relative to crypt height at which cells stop proliferating	set manually to match qualitative data from the literature [4]	1776^a^
proliferation boundary	position relative to crypt height of switch between stem and proliferating cells	set manually to match qualitative data from the literature [10]	296^a^
attachment strength	the strength of the cell binding to the basement membrane	range determined by parameter tuning (see Results section)	0.01–0.0001^b^

^a^Distances in the model are expressed in arbitrary units as all scales are calculated relative to crypt radius.

^b^Unitless parameter.

### 2.3. Process overview and scheduling

**Figure 2.**
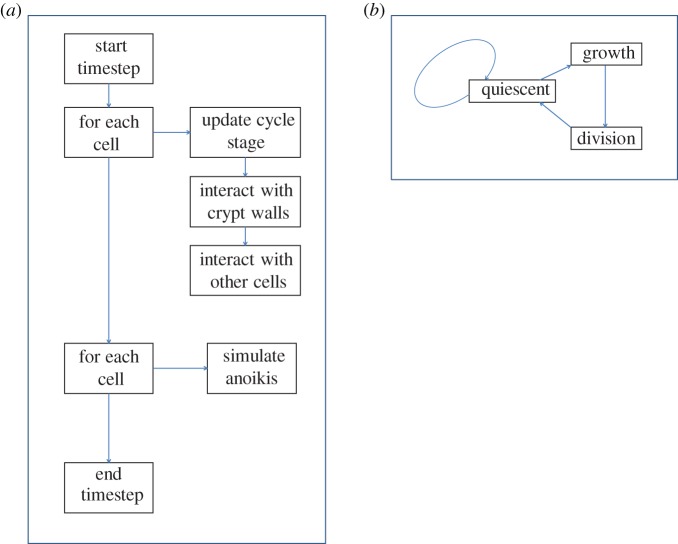
(*a*) Flow chart showing a broad overview of the update process at each timestep of the model. (*b*) Flow chart showing the model cell cycle.

The model updates in a series of discrete timesteps, each representing 30 seconds of biological time. At each timestep a series of separate rules are applied (figure 2*a*).

### 2.4. Design concepts

#### 2.4.1. Basic principles

The model is an agent-based model. Each cell is represented by a software agent which behaves autonomously with no overarching control mechanism, each agent displaying a number of biologically based behaviours.

#### 2.4.2. Movement

Movement and division of cell agents results in collisions. Detected overlaps are corrected by applying forces based on the concept of cell centres connected by stiff springs (box 1). These forces are modelled by equation (2.1):

2.1 $$\text{F}\;=(r(i)+r(\;j)-\;|\text{x}(t,i)-\;\text{x}(t,j)|)\times \frac{\text{x}(t,i)-\;\text{x}(t,j)}{\left|\text{x}(t,i)-\text{x}(t,j)\right|}\times \;a,$$

where **x**(*t*, *i*) is the position of cell *i* at iteration *t*, *r*(*i*) is the radius of cell *i*, cell *i* and cell *j* are the overlapping cells, and *a* is a constant representing the spring stiffness. This has been shown to be a valid method to represent cell–cell interactions in tissue simulations [26] and has been used in colorectal crypt simulations since the earliest agent-based models [16]. This equation is solved using Euler integration. The equation is applied in the plane tangential to the basement membrane at the point where the cells contact. This causes the cells to behave as cylinders rather than the more commonly modelled spheres, as a cylinder better represents the shape of cells seen in *in vivo* crypt sections [27].

Box 1. **Overlap resolution rule.**

For every other cell

 If cell overlaps other cell

  Calculate restoration force based on equation (2.1).

  Apply restoration force to both cells

#### 2.4.3. Crypt geometry

The crypt shape is modelled as three conjoined mathematical surfaces, a hemisphere, an open cylinder and a quarter-torus (figure 1*b*), to which the cells are attached by a damped spring force rule based on the collision resolving force rule. This rule represents a spring, with rest length 0, between the cell centre and the closest point on the basement membrane (box 2).

Box 2. **Membrane attachment rule.**

Find closest point to cell on membrane

Move cell towards membrane point

#### 2.4.4. Cell cycle

The cell is modelled as a finite state machine undergoing transitions between a pre-defined number of states which represent the phases of the cell cycle (figure 2*b*). A cell has one of three types determined by its location within the crypt (figure 1*c*, table 2): differentiated (red), proliferating (blue) or stem (purple). Not all cells are in cycle at all times. Differentiated cells never enter the cell cycle. Proliferating cells re-enter the cell cycle immediately after division. Stem cells enter a quiescent phase after division before re-entering the cell cycle. This corresponds to the niche hypothesis of crypt organization [6]. Cell division at the end of the cycle is modelled by growing a new cell from zero size inside the dividing cell (box 3, figure 1*d*), an approach which prevents artefacts arising from the sudden force associated with the instantaneous appearance of two full sized cells. This ensures that by the end of the mitosis phase the new cell is located adjacent to the original cell on a random bearing and that it is fully integrated with the surrounding cells in terms of contact forces. The random bearing of the new cell is not necessarily in the plane of the basement membrane but the membrane attachment force will rapidly move it into this plane. See boxes 4, 5 and 6 for pseudocode describing the rules governing the cycle stages.

Box 3. **Quiescent phase rule.**

If cell is quiescent

 If cell has been quiescent longer than G0 time

  Cell enters G1

Box 4. **Growth phase rule.**

If cell is growing

 If cell has been growing longer than G1 time

  Create child cell

  Cell enters M phase

 Else

  Cell gets bigger

Box 5. **M phase rule.**

If cell is in M phase

 If cell has been mitotic longer than M time

  Child cell splits off

  Cell enters G0

Else

  Child cell gets bigger

  Cell gets smaller

Box 6. **Simulation boundary rule.**

If cell is beyond simulation boundary

 Move cell towards simulation boundary

#### 2.4.5. Simulation boundary

Cells which pass beyond the simulation boundary have a spring-based restoring force applied to them which causes the boundary to act as a mirror of the model crypt, producing the same result as if the crypt had neighbouring crypts on all sides as seen in the biology (i.e. reflective boundary conditions are imposed). See box 6 for more details.

#### 2.4.6. Anoikis

Our model includes the process of anoikis whereby if a cell moves more than a certain threshold distance, defined in table 2, from the basement membrane, it is considered to have lost contact and cell death is triggered (box 7).

Box 7. **Anoikis rule.**

Find closest point to cell on membrane

If distance from cell to membrane is greater than anoikis threshold

 Kill cell

### 2.5. Stochasticity

Stochasticity is introduced into the model by varying the cell cycle time per cell. Every time a cell divides, the two daughter cells are designated values of cell cycle times that are sampled from a normal distribution with mean and standard deviation extrapolated from *in vivo* data [10].

### 2.6. Output

The overall cellularity of the crypt was tracked throughout the simulation. The rates of anoikis and division events were recorded. The position of each anoikis event relative to crypt height was also recorded.

### 2.7. Access to code

The code generated and used in virtual experiments is available at the Dryad Digital Repository: http://datadryad.org/review?doi=doi:10.5061/dryad.800pj [28].

### 2.8. Initialization

The model is initialized as a single empty crypt. This crypt is uniformly filled with cells spaced at two cell radii apart. Cells are not placed closer than this to avoid generating large forces due to over-packing. The initial type of each cell depends on its location in the crypt according to the niche hypothesis. The time of the first division event for each cell is initialized independently to a value drawn from a uniform random distribution. This results in a realistic distribution of cell cycle phase throughout the crypt.

### 2.9. Virtual experiments

A number of virtual experiments were carried out to investigate homeostatic processes within the model crypt. Each simulation was run for approximately 1 year of simulated biological time and was repeated ten times with results being analysed as mean and standard deviation of these repeats to account for stochasticity.

## 3. Results

### 3.1. Anoikis localizes to the crypt mouth as an emergent behaviour

The mean cell cycle time was set to 30 h. Other parameters are as recorded in table 2. For each simulation, the position of anoikis events within the crypt was recorded and distributed into a series of 100 bins, each one representing 1% of crypt height. The mean and standard deviation of the total number of events in each bin was calculated over the ten runs.

Our model includes migration and membrane attachment rules, but does not formally direct anoikis events, rather they occur emergently as a response to local conditions within the crypt. This simulation series aimed to establish the predicted location of anoikis in the model crypt and used ten repeat simulations to account for stochasticity.

**Figure 3.**
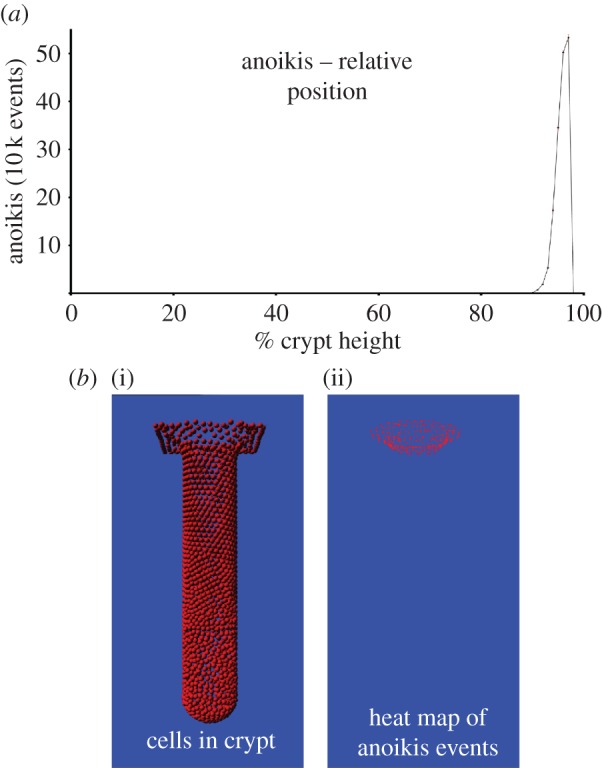
(*a*) Graph of anoikis locations in the model crypt. (*b*(i)) Image of cells within the model crypt for comparison to (*b*(ii)), ashowing heat map of anoikis locations during a simulation. These results are from ten repeat simulations with a cycle time of 30 h and attachment parameter of 0.001.

Figure 3*a* shows that anoikis is localized to the top 5% of the crypt. As can be seen by the small error bars there is very little variation across different simulation runs. Figure 3*b*(ii) visualizes the anoikis events localizing to the upper region of the crypt on a heat map on a geometrical representation of the crypt. Comparison with the crypt visualization in figure 3*b*(i) shows that events are confined to the curved area at the mouth of the crypt. This experiment was re-run with different cell cycle times and attachment forces all of which produced identical localization of anoikis. These results are provided in the electronic supplementary material. Mean lifetime of a differentiated cell was 3.4 days, which is biologically realistic [25]. The cells in the crypt displayed migratory behaviour, moving steadily from the crypt base to the crypt mouth with a mean migration velocity of 0.73 cell radii per hour, which also reflects biological results [25].

### 3.2. Anoikis rate is a potential determinant of cellular homeostasis at the crypt level in response to alteration in proliferation

**Figure 4.**
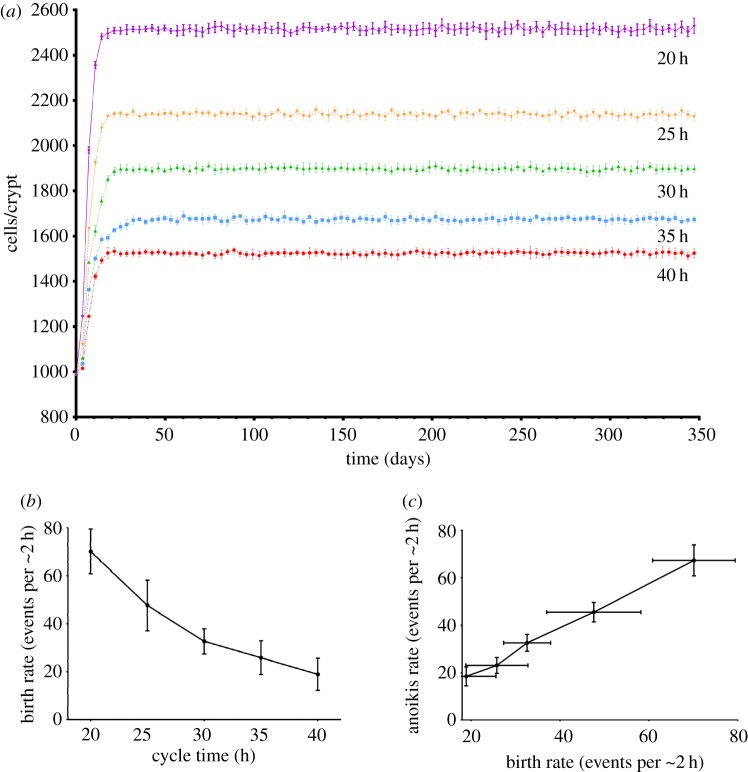
(*a*) Graph of number of cells in crypt over time for different cell cycle times. (*b*) Graph of birth rate against cycle time. (*c*) Graph of birth rate against anoikis rate for this set of simulations.

Model simulations were undertaken to assess the impact on crypt cellularity of altered lengths of cell cycle. The model was run ten times with mean cell cycle times of 20, 25, 30, 35 and 40 h for each set of simulations. The total cell number, number of birth events and number of anoikis events were recorded every 200 timesteps. The mean and standard deviation of each set of data produced across the ten replicates were calculated and results are presented in figure 4. With a biologically relevant range of cell cycle times of 20, 25, 30, 35 and 40 h, the crypt reaches equilibrium in all cases (figure 4*a*).

Birth rates (defined here as number of cell division events in a two hour time period) increase as cycle time reduces, as might be expected (figure 4*b*). This shows a snapshot of birth rates taken at a single time point across all ten runs. The chosen time point was equivalent to approximately six months of biological time since the start of the simulation to ensure that equilibrium had been reached. The increase in birth rate as cycle time reduces emerges partly as a result of the rules governing cell division. However, the rules alone would only account for a linear increase. The greater than linear increase is a non-intuitive result and is discussed further below.

Figure 4*c* shows that anoikis rates (events per two hours) quantitatively match birth rates, which causes the model to reach equilibrium and is a non-enforced emergent phenomenon. This is again a snapshot taken at the six month point across all runs. Matching of birth rates is an emergent phenomenon and not encoded in the rule-set of the model.

### 3.3. Membrane attachment force influences cellularity of the crypt

**Figure 5.**
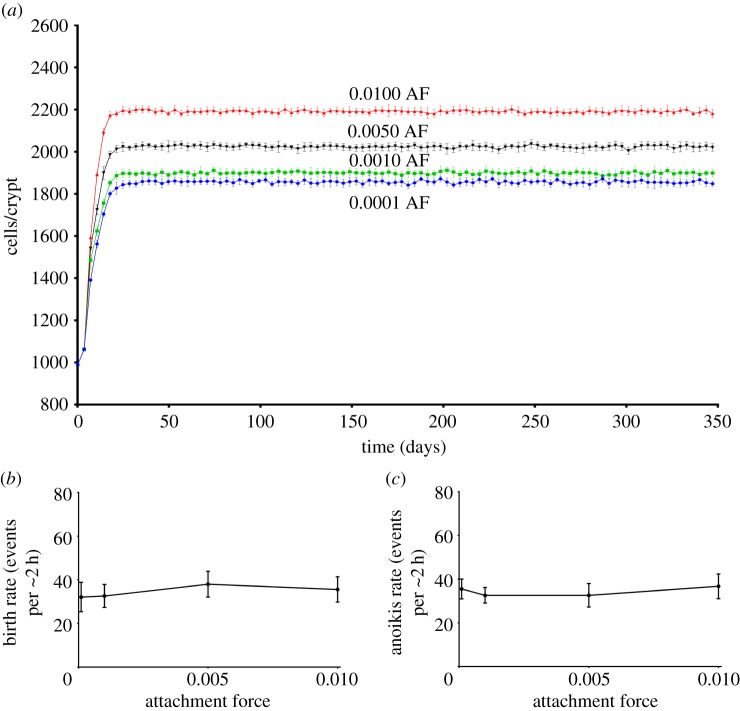
(*a*) Graph of number of cells in crypt over time for different membrane attachment values (AF). (*b*) Graph of birth rate against attachment value. (*c*) Graph of anoikis rate against attachment value.

During the earliest stages of carcinogenesis, mutation of APC is hypothesized to lead to elevated attachment between the cell and the basement membrane; this occurs through derangement of the β-catenin role [29]. We sought to investigate the consequence of altered attachment force on crypt cellularity. The model was run ten times with a mean cycle time of 30 h. As absolute attachment force values are not available, we chose to select parameter values across a three-log range of 0.01, 0.001, 0.005 and 0.0001, with 0.001 being the default value in our model. Figure 5*a* shows that increasing the attachment force increases the equilibrium cellularity level. In order to investigate the basis of this effect, we assessed the impact of change in attachment force parameter upon birth rate and anoikis. Figure 5*b* shows that increasing the attachment force parameter does not increase the birth rate. Figure 5*c* shows that, again, anoikis rates adapt to match birth rates for the range of attachment forces modelled. This is also an emergent phenomenon that cannot be attributed to any single underlying rule.

## 4. Discussion

We have built an ABM to explore interrelationships between cell cycle rate, attachment force, crypt morphology and homeostasis. We show that anoikis emerges in the regions of the crypt where it is primarily observed *in vivo* [30]. In addition to this, the localization of anoikis to the crypt top causes the model to exhibit the emergent migration of cells documented *in vivo* [7], without the presence of any apoptotic signal at the crypt top. It is possible that such signalling exists *in vivo*, alongside the apoptosis mechanism modelled here, and the relative impact of the two mechanisms is an open question. There are no data in the literature seeking to clarify the balance of active signalling and passive attachment force in modelling anoikis. The predicted localization of anoikis may be due to the negative curvature at the top of the crypt which causes the lateral forces the cells exert on each other to be directed away from the membrane, in turn pushing the cells off the membrane in this, and only this, region of the crypt. This varies from the predictions of previous models in which anoikis did not correctly localize to the crypt top [20]. The difference is accounted for by the more accurate cell geometry used in this study. When the model presented here is run with spherical, rather than cylindrical, cells, anoikis no longer localizes to the crypt top (see electronic supplementary material).

The second series of simulations suggested that anoikis regulates crypt cellularity. In the simulation, cellularity always reached equilibrium, even without an imposed, signalling-based apoptotic mechanism (although, as mentioned above, such a mechanism is not ruled out *in vivo*). The simulation shows that the model contains an emergent regulatory mechanism whereby increased proliferation drives a compensatory elevation of anoikis. This occurs because higher cellularity causes greater compression of the cells, which in turn causes higher forces, pushing the cells off the membrane more rapidly. This feedback mechanism raises interesting questions for biological study, as it suggests that hyperproliferation alone would not be a sufficient cause of neoplasia, and that other alterations (mutations or pleiotropies) permitting progressive acquisition of excess cells would need to be invoked. This is in agreement with biological studies which have suggested that anoikis could act as a homeostatic mechanism and frequently occurs for non-apoptotic cells [13].

It may seem that the balancing of anoikis and birth rate is an expected outcome since the geometry cannot expand to incorporate new cells. However, while it is true that the geometry is fixed, the cells are able to compress such that if no coupling emerged between anoikis and birth rate then the cells may eventually compress to zero size. This would be biologically unrealistic but could be interpreted as a trigger for biological phenomena that the model in its current form cannot explicitly represent, for example crypt fission or the formation of an adenoma. This model does not reach such a state due to the tracking of anoikis to birth rate, which is not an explicit rule of the model but is emergent. This coupling is a consequence of the linking of cellularity to anoikis rates through compression forces. It is not a foregone conclusion that such a coupling will arise in the model as over most of the crypt the forces generated from cellular compression are either orthogonal to or opposing the forces required for anoikis; it is only in the crypt mouth that these forces align and if this was not the case there would be no coupling between birth rate and anoikis rate.

Our simulations also show that birth rates (where birth rate is the total number of divisions per two hour period, and hence is dependent on the size of the cell population) increase when cell cycle times are reduced. This is a result expected to arise from the rules implemented in the model. However, the amount of increase observed is exponential (figure 4*b*), in contrast to a linear increase that would naively be expected. This emergent behaviour can be accounted for as follows: reducing the cycle time increases the birth rate and thus indirectly the total cellularity of the crypt. This additional cellularity is contained within the crypt by the cells becoming more compressed and hence more closely packed. This means that, in line with the assumed niche hypothesis, any fixed size portion of the crypt, including the proliferative compartment, will have more cells in it as a consequence of higher cellularity, leading to more cells being in cycle at any one time. This accounts for the additional increase in birth rates in the model. It has been observed *in vivo* that the proliferative compartment expands in proximity to a lesion [31]. This may be due to increased pressure being exerted on the healthy crypt from neoplastic neighbours due to their higher rates of proliferation and lower rates of anoikis. This increased pressure might compress the healthy crypt and so force more cells into the proliferating compartment (again, assuming the niche hypothesis). The model could be used to investigate this phenomenon further in the future. An interesting feature of the cycle time simulations was that increased cell turnover led to increased crypt cellularity even though death rates rose to match increased birth rates. This is because the increased death rates were a result of greater compression forces in the crypt. As such, higher cellularity is required to achieve this greater compression and cause death rates to catch up to birth rates. Attachment strength affects cellularity in a similar manner: greater compression is required to overcome the greater attachment strength and induce anoikis.

We explored the consequences of varying the parameter representing the strength of attachment between colonocytes and the underlying basement membrane. As attachment is modelled using the same damped spring model as cell–cell overlap resolution, attachment strength is represented by a spring stiffness parameter which cannot directly translate to specific values of attachment force. It does, however, allow the model to make qualitative predictions of the behaviour resulting from stronger and weaker attachments. By regulating how strongly the cells are attached to the basement membrane, the parameter therefore influences how much they resist anoikis. Increased adhesion is a potential feature of cells with APC loss [29]. This parameter is dependent *in vivo* on a number of factors (e.g. β-catenin localizing to adherens junctions [29]) but actual forces have not been measured quantitatively. A stronger attachment requires a greater force to achieve the same rate of anoikis, resulting in higher cellularity in the model. The forces acting on the cells are driven by the level of cell compression, which is in turn driven by cellularity. This means that a higher cellularity is required in order for anoikis rate to match a given birth rate.

By explicitly resolving interactions between cells and cells, and cells and the basement membrane, this model offers a distinct advantage to compartmental and continuum models for understanding the mechanical processes within the crypt, and furthermore extends the application of previous ABMs by extending the rules governing cell death to better reflect the underlying biology. The model might be used to discriminate between different hypotheses for mutation embedding. By incorporating a region of flat mucosa the model extends the spatial extent of the biological region represented. Future developments will include upscaling the model to simulate multiple interacting crypts, and extending the cell model to reflect the effects of known pre-cancerous mutations.

## 5. Conclusion

A new computational model of the colonic crypt has been developed. Unlike previous crypt models, the geometry of the crypt top has been included, as well as the explicit mechanism of attachment related cell death, or anoikis. In agreement with published biological observations, our results predict the localization of anoikis events exclusively to the top of the crypt. These results also suggest that anoikis plays an important role in regulating crypt cellularity against changes in proliferation and membrane attachment. This suggests a potentially chemo-preventive function for this previously unknown mechanism, which may be a worthwhile focus for future *in vivo* study.

## Supplementary Material

Additional Simulations

## Data accessibility

Code generated and used in this paper is available at the Dryad Digital Repository: http://datadryad.org/review?doi=doi:10.5061/dryad.800pj [28].
