# Supplementary material for: An agent-based model of anoikis in the colon crypt displays novel emergent behaviour consistent with biological observations
Source: R Soc Open Sci. 2017 Apr 12;4(4):160858. doi: 10.1098/rsos.160858 (PMC5414243; doi:10.1098/rsos.160858)
Supplement: Additional Simulations [file rsos160858supp1.pptx]

## Slide 1
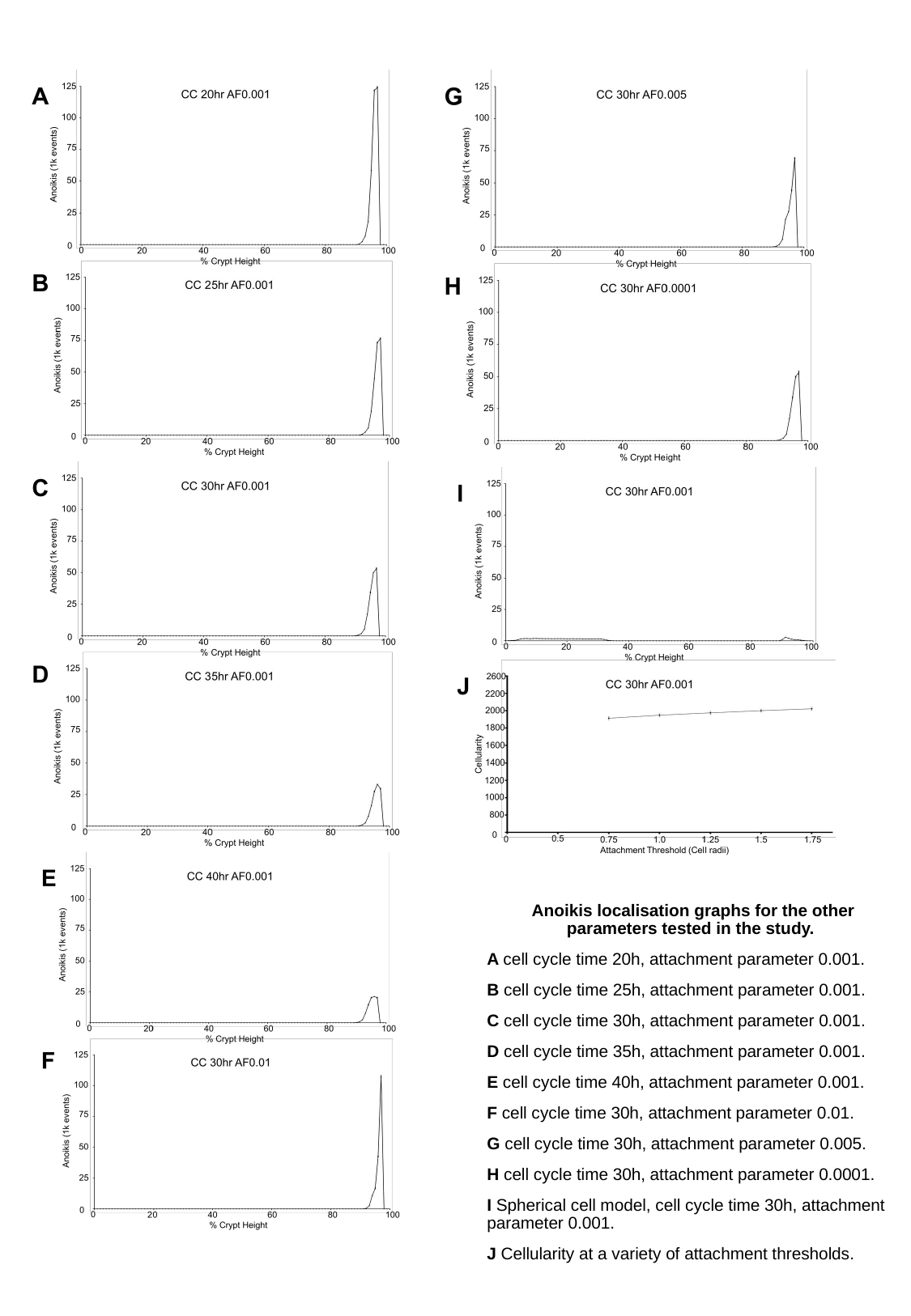

#
Anoikis localisation graphs for the other parameters tested in the study.
A cell cycle time 20h, attachment parameter 0.001.
B cell cycle time 25h, attachment parameter 0.001.
C cell cycle time 30h, attachment parameter 0.001.
D cell cycle time 35h, attachment parameter 0.001.
E cell cycle time 40h, attachment parameter 0.001.
F cell cycle time 30h, attachment parameter 0.01.
G cell cycle time 30h, attachment parameter 0.005.
H cell cycle time 30h, attachment parameter 0.0001.
I Spherical cell model, cell cycle time 30h, attachment parameter 0.001.
J Cellularity at a variety of attachment thresholds.
